# Supplementary figures and images for: An affordable and automated imaging approach to acquire highly resolved individual data—an example of copepod growth in response to multiple stressors
Source: PeerJ. 2019 Apr 19;7:e6776. doi: 10.7717/peerj.6776 (PMC6476288; doi:10.7717/peerj.6776)

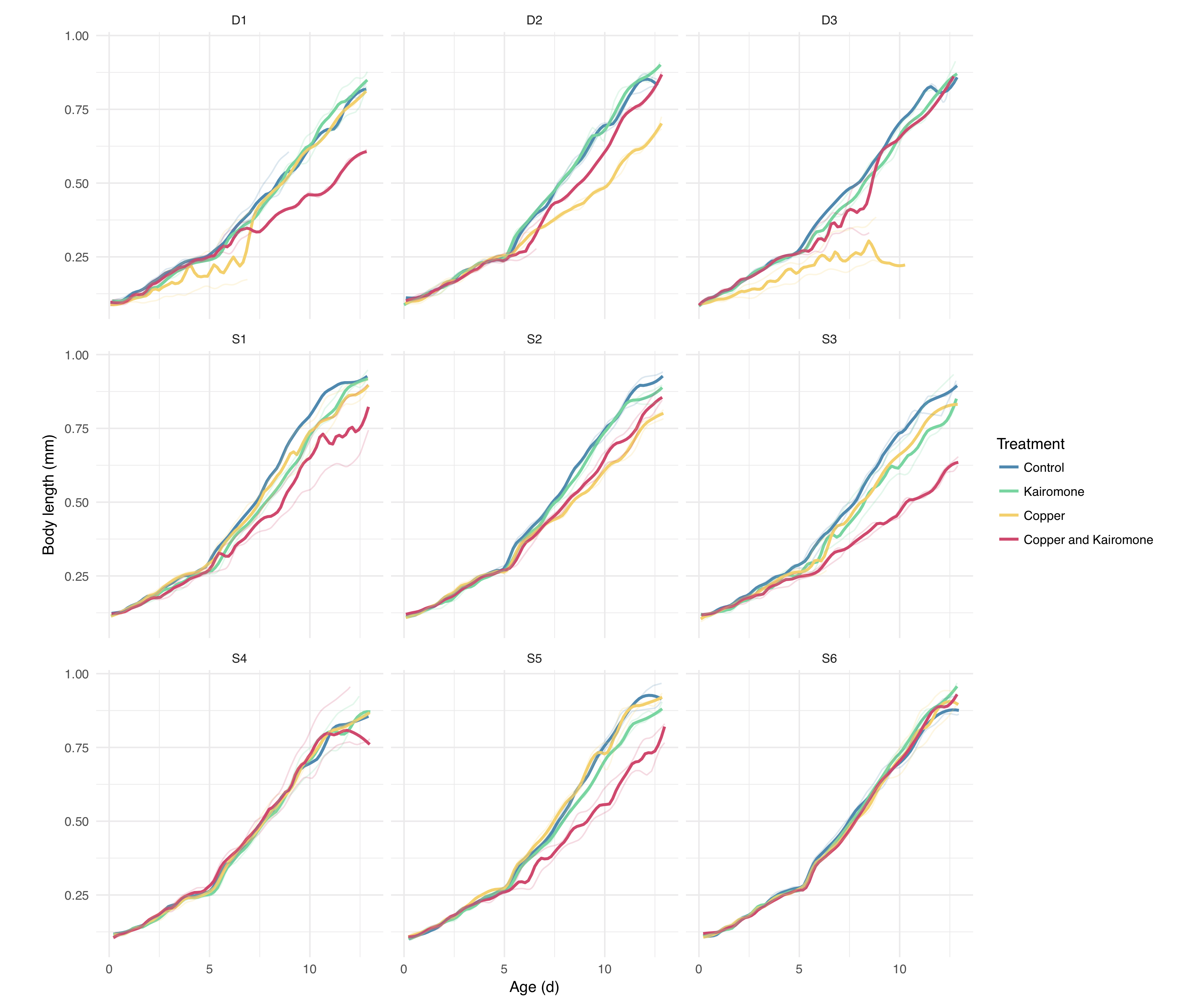

Supplement: Supplemental Information 3 — Faint lines indicate individual growth trajectories, while bold lines show the final GAM predictions averaged by treatment. [file peerj-07-6776-s003.png]
